# Active viscoelastic models for cell and tissue mechanics

**DOI:** 10.1098/rsos.231074

**Published:** 2024-04-24

**Authors:** Bahareh Tajvidi Safa, Changjin Huang, Alexandre Kabla, Ruiguo Yang

**Affiliations:** ^1^ Department of Mechanical and Materials Engineering, University of Nebraska-Lincoln, Lincoln, NE 68588, USA; ^2^ School of Mechanical & Aerospace Engineering, Nanyang Technological University, Singapore 639798, Singapore; ^3^ Department of Engineering, University of Cambridge, Cambridge CB2 1PZ, UK; ^4^ Department of Biomedical Engineering, Michigan State University, East Lansing, MI 48824, USA; ^5^ Institute for Quantitative Health Science and Engineering (IQ), Michigan State University, East Lansing, MI 48824, USA

**Keywords:** active model, viscoelasticity, cell modeling, cell mechanics, tissue mechanics

## Abstract

Living cells are out of equilibrium active materials. Cell-generated forces are transmitted across the cytoskeleton network and to the extracellular environment. These active force interactions shape cellular mechanical behaviour, trigger mechano-sensing, regulate cell adaptation to the microenvironment and can affect disease outcomes. In recent years, the mechanobiology community has witnessed the emergence of many experimental and theoretical approaches to study cells as mechanically active materials. In this review, we highlight recent advancements in incorporating active characteristics of cellular behaviour at different length scales into classic viscoelastic models by either adding an active tension-generating element or adjusting the resting length of an elastic element in the model. Summarizing the two groups of approaches, we will review the formulation and application of these models to understand cellular adaptation mechanisms in response to various types of mechanical stimuli, such as the effect of extracellular matrix properties and external loadings or deformations.

## 1. Introduction

Living cells, their surrounding extracellular matrices (ECM) and tissues as a whole exhibit viscoelastic properties, that is, having both an elastic and a viscous behaviour. Mechanical tests are used to characterize the mechanical properties of cells and tissues and help us understand and predict cellular behaviour in healthy or pathological conditions at different time scales [1–4]. For example, experimental studies on cell monolayers devoid of substrate have reported a viscoelastic solid-like behaviour under constant strains [5]. In other words, on the time scale of seconds, cell monolayers behave like a viscous fluid and dissipate stress, then reach a plateau in stress on the time scale of minutes, which is a characteristic of elastic solid materials [5]. On the other hand, cell aggregates exhibit solid-like behaviour on short time scales, for example, in a few seconds, and fluid-like behaviour at time scales of the order of minutes to hours [4,6,7]. In this case, the behaviour of cell aggregates is similar to a viscoelastic fluid material. Additionally, the mechanisms governing the viscous-like behaviour can also differ depending on the time scale of load application [8]. For example, on the time scale of tens of seconds to minutes, stress dissipation occurs owing to the turnover of actin filaments and reorganization of the actomyosin network [4,8,9]. On the time scale of minutes to hours, stress dissipation mechanisms at the cellular scale, such as oriented cell division and cell rearrangements, can start to influence the response to mechanical loadings [8,10–12]. Transitions between fluid-like and solid-like behaviours in living cells can also occur in response to mechanical stimuli. For instance, short-term fluidization has been reported immediately upon strain application, which is usually followed by stiffening [13–16].

The experimental results are often analysed by mathematical models to capture the important features of the material response, presented in terms of model parameters. These parameters can then be used for classification, comparison and prediction of the mechanical behaviour of cells and tissues subjected to other loading conditions [17]. By relating the model parameters to the underlying biological processes on the molecular and cellular scale, the physical meaning of the parameters can sometimes be assigned. Since tissues exhibit time-dependent mechanical behaviours [5,18], a common modelling approach is to consider the cells and tissues as a viscoelastic continuum and to describe their mechanical response from quantitative mechanical interrogations, often stress–strain relationships, in terms of a combination of stiffness and viscosity, or elastic and loss moduli under dynamic loadings. These extracted mechanical properties have long been regarded as disease biomarkers [19,20]. For instance, metastatic cancerous cells exhibit lower stiffness than benign cells [21].

Perhaps more importantly, cell mechanics is not only a by-product of the underlying molecular structure but also a means for cells to actively adapt to environmental cues, in service to a preferred cellular function, such as cell migration in wound healing [22] and tissue morphogenesis in development [23]. Being viscoelastic in nature, cells can dissipate the imposed stress owing to external strains. However, sometimes this passive response is insufficient to maintain the mechanical integrity of the cell. For instance, cells need to use effective stress relaxation mechanisms such as actin polymerization to prevent tissue fracture. In other instances, under rapid strain applications, cells may need to stiffen or actively pull back to make further deformation difficult, thus preventing further damage to their cytoskeleton. From the adaptation perspective, the evolving elastic and viscous properties owing to the active adjustments of cytoskeleton tension can be considered as a way to facilitate the response and adaptation to external stimuli. Probing active cellular behaviour through the lens of mechanics is particularly intriguing because it offers a window from which the adaptation can be quantitatively examined up close with defined mechanical stimuli.

In the cell cortex as well as at the cell population scale, living matter not only responds to external forces or deformation as any traditional material would but also often exhibits force-generating mechanisms emerging from actin polymerization, adhesion dynamics and actomyosin contractility. This process is referred to as being ‘active’ in this review. Active matter theory is another continuum-based model used to describe the dynamics of cell cytoskeleton and cell monolayers (reviewed in [9,24–26]). This type of model is based on the theory of liquid crystals and can be employed to describe the mechanics of the actomyosin cortex for time scales longer than the turnover time of actin filaments [27]. However, these models rarely capture the way such materials respond to external deformations or stresses; it remains challenging to capture, both experimentally and theoretically, the impact of active processes on the mechanical state of living matter.

**Figure 1.**
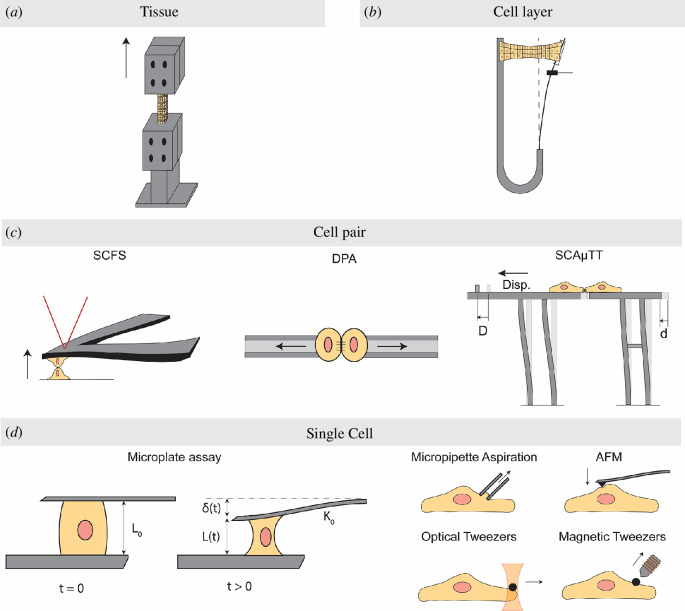
Experimental techniques used to probe cell mechanics in various spatial scales. (*a*) Mechanical testing machine used to study the response of tissue samples in compression and tension tests. (*b*) The recent technique developed for investigating the mechanical response of cell layers to uniaxial stretch and compression. (*c*) In cell pair studies, mechanical stimulus is applied through various methods such as a single cell adhered to a tipless atomic force microscopy (AFM) cantilever in single-cell force spectroscopy (SCFS), two micropipettes in dual pipette aspiration (DPA) and a microstructure fabricated using two-photon polymerization technique in single-cell adhesion micro tensile tester (SCA μ TT). (*d*) Microplate assay is a commonly used method to monitor the force generated by individual cells when exposed to variations in substrate stiffness. Other techniques, such as micropipette aspiration, AFM and optical and magnetic tweezers, involve applying controlled displacement as the mechanical stimulus and monitoring the forces within cells or investigating changes in displacement in response to controlled forces.

Experimentally, a common approach is to subject the cellular materials to tension or compression and then assess their response using stress–strain curves. At the tissue scale, mechanical testing machines can be used to investigate the macroscopic behaviour of tissue samples (figure 1*a*) [28,29]. A similar technique has been developed to study the response of cell monolayers when subjected to compression or tension (figure 1*b*) [30]. In addition, to examine the microscale characteristics of living tissues, atomic force microscopy (AFM) and nanoindentation techniques can be employed, where a probe tip is used to apply forces in the pico-nano Newton range [31,32].

The AFM technique can also be used to study the active and passive behaviour of individual cells [33–35] and can be adapted for cell pair studies in single-cell force spectroscopy (SCFS), where a single cell adhered to the cantilever beam serves as the probe tip (figure 1*c*) [36]. Furthermore, various methods are developed to study the response of single cells and cell doublets in tension and compression tests. For instance, in microplate assays, cells are compressed or stretched between a fixed and a deformable plate [37,38], microbead assays use microbeads to apply force via magnetic [39,40] or optical tweezers [41–43] (figure 1*d*), and the micropipette aspiration method involves subjecting cells to negative pressures and measuring their deformation [44–46] (figure 1*d*). This method can also be used for cell pair studies by bringing two cells into contact via micropipettes, that is, dual pipette aspiration (DPA) (figure 1*c*) [47], or it can be combined with other techniques such as optical tweezers [48]. Recently, a new micromanipulator device has been introduced that can directly measure forces in cell pairs under controlled loading conditions, leading to advancements in the precision of the interrogation of cell pair mechanics (figure 1*c*) [49]. Different techniques have varied ranges of resolution and loading rates; thus, one has to consider the application requirements when choosing an experimental technique. For instance, AFM techniques benefit from higher spatial resolution and force sensitivity compared with methods employing micropipette aspiration [50,51].

These experimental techniques are employed to probe cellular response at various temporal and spatial scales. In addition to measuring the global mechanical behaviour of cells and tissues, these techniques can also be employed to examine the mechanical properties of specific components of cells. For example, micropipette aspiration techniques have been used to investigate the microrheology of the cell nuclei [52–54]. The monitored stress/strain response of the cellular materials will be the outcome of both active force-generating and passive mechanisms operating at those scales. From a modelling perspective, capturing active behaviour requires a mechanism of introducing change to the otherwise fixed viscoelastic models. Researchers over the years have incorporated active adaptation mechanisms by introducing active empirical mathematical models to link mechanical characteristics to the underlying biological processes without simulating the details of the underlying chemical signals. Rheological models for describing the mechanical behaviour of cellularized materials across various length scales have previously been reviewed [4,15,17,55]. In this article, our primary goal is to focus on different forms of integrating activity into rheological models, emphasizing variations in their definitions. We here summarized these reported mechanisms into two broad categories. The first group integrates a force/stress (force divided by cross-sectional area)-generating element to classic viscoelastic models. This active element can be constant or time-dependent. The second group introduced mathematical methods that adjust the resting length of an elastic spring or the reference stress-free shape in two dimensions/three dimensions in classic viscoelastic models. Mathematical models in both categories are developed in conjunction with specific techniques that probe the active cell response to various types of mechanical stimuli. In some cases, the active models can be mathematically equivalent, but different parameters may offer a better link with the underlying biology. In this article, we present a selection of such models, introducing first the experimental findings and then focusing on the formulation and application of each type of active viscoelastic model. We will begin with a short introduction to the biology of active cell behaviour.

## 2. Biology of cellular active behaviour

Cell cytoskeleton mainly comprises filamentous proteins that preserve the cell structure, arrange organelles, and resist, transmit and generate forces [56,57]. These proteins can be categorized into three groups: microtubules, intermediate filaments and actin filaments [58]. Active force generation is realized by actin filaments. They are constructed by assembling monomeric actin. Actin-binding proteins bind to actin filaments and form different structures, such as the lamellipodium network, contractile bundles of stress fibres and the contractile network of the cell cortex [57,59]. Actin filaments are engaged in active processes such as actin treadmilling and force generation by consuming the energy provided by adenosine triphosphate (ATP) hydrolysis [60–62]. ATP molecules attach to ATP binding sites on actin monomers, and the ATP-bound actin monomers will be assembled at the plus/barbed end of the actin filaments leading to filament growth [60,63]. ATP molecules will slowly hydrolyse to adenosine diphosphate (ADP) and the ADP-bound actin monomers will start to disassemble from the minus/pointed end of the actin filaments [60,64]. The process of assembling and disassembling actin monomers is referred to as actin treadmilling [65]. Actin filaments use the energy from ATP hydrolysis to generate protrusion forces to help cells in spreading and migration [66,67]. Actin treadmilling is also crucial in endocytosis, exocytosis and phagocytosis to engulf large particles [57].

In addition, ATP hydrolysis provides the energy for contractile stress generation in the actomyosin network. Myosin motors convert chemical energy from ATP hydrolysis to mechanical energy and slide actin filaments past one another to produce force [68–70]. This process is similar to the shortening of sarcomeres in muscle cells [37,38,64,70]. The contractile forces generated from this process will then be transmitted to neighbouring cells and the ECM via cell–cell junctions and focal adhesions, respectively [66,71,72]. At the cell and tissue level, contractile force production controls cellular activities, such as cell migration [67,73–75], proliferation [76,77], stem cell lineage determination [78,79], tissue regeneration [80] and morphogenesis [81,82]. Important for cellular mechanical characterization, contractile forces also regulate cellular response to substrate stiffness and mechanosensing [83]. The active ATP-dependent processes that are at play in subcellular scales can also regulate cellular response under various loading conditions at cell and tissue scales [5,59,84,85].

## 3. Active contractile elements

Rheological models are useful tools to quantitatively analyse the results of mechanical tests. However, associating the molecular scale origin with the model parameters is challenging, in particular, where cell behaviour deviates from the response of traditional passive materials [17]. Thus, adding elements that represent the active behaviour of cells can equip classic models with the ability to empirically link the mechanical response to the underlying biological processes.

To represent active cellular dynamics from intracellular contractility, contractile/force-generating elements (CEs) can be added to standard viscoelastic models. In this section, we will discuss active CEs that can mimic cell behaviour when they are exposed to different types of mechanical stimuli. We begin with the first active model, which was presented by Hill to elucidate muscle contraction and its application in studying mechanosensing in single cells. In the following section, we will explore an active fluid model that considers the transient nature of the actin network to describe characteristics of the single-cell response to the substrate stiffness. In this model framework, tension generation via myosin activity will be denoted by $\sigma_{a}$ , and regulating mechanisms of the dynamics of tension build-up will be discussed. Additionally, we will also provide some examples for both models.

### 3.1. Muscle contraction defined by the Hill model

**Figure 2.**
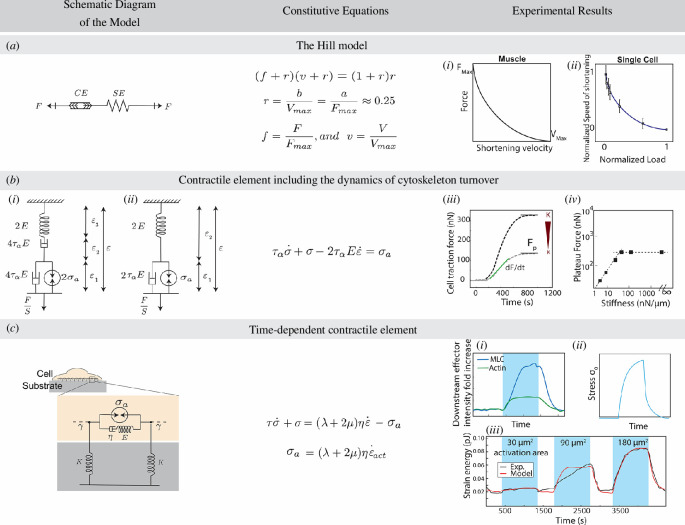
Models of active contractility relating force/tension generation and the rate of contraction. (*a*) Model proposed by Hill [29] to describe muscle contraction [86]. CE serves as a force-generating contractile element and SE is an elastic spring that affects the dynamics of contraction. (**i**) Force–velocity curve obtained in Hill’s experiments. (ii) Normalized speed of shortening $\left(v=V/V_{max}\right)$ and normalized force generation $\left(f=F/F_{max}\right)$ in single cells in response to various substrate stiffnesses can be represented by the reduced Hill equation (3.2) [37]. (*b*) Active fluid model proposed to study mechanotransduction in cells [38]. (i) This model accounts for the transiently cross-linked nature of the actomyosin network and force generation via myosin motor activity. (ii) Equivalent of the model presented in (i), which has the same constitutive equation. The net effect of two dashpots is similar to one dashpot. (iii) Evolution of traction force generation over time for low (grey) and high (black) substrate stiffness. The two main characteristics of force generation–time curves, that is, the plateau force $F_{p}$ and the rate of force generation (dF/dt) are dependent on the substrate stiffness [37]. (iv) The plateau force has a linear relationship with substrate stiffnesses when the stiffness is below 60 nN μm^−1^ and for higher values of stiffness, $F_{p}$ saturates [37]. (*c*) Active Maxwell model that assumes a time-dependent increase in cellular contractile force with the same profile as actin and myosin accumulation in the RhoA-activated areas [87]. (**i**) Changes in the local intensity of actin and myosin in the regions of RhoA activation. (ii) The plateauing exponential function used in the model to represent the stress profile during activation of RhoA. (iii) Experimental (black) and theoretical (red) evolution of strain energy during intervals of RhoA activation and relaxation.

Muscles function as intricate biochemical mechanisms that transform chemical energy into mechanical energy through actomyosin interactions to provide movement in our bodies. Hill proposed the first theory to describe muscle contraction back in 1938 [29]. He conducted experiments using the sartorius muscle of a frog and explained the macro properties of muscles. In these experiments, Hill quantified both force generation and velocity of length changes in muscles under various loading conditions. The two endpoints of the curve representing the force–velocity relationship in muscles (figure 2*a*(i)) were generated in two extreme cases: zero force (isotonic condition) and zero velocity (isometric condition). Specifically, when force is kept at 0, the muscle can reach its maximum shortening velocity, $V_{\text{max}}$ . Similarly, when the muscle is restricted from changing its length, the maximum level of force, that is, the stall force $F_{\text{max}}$ , can be generated at the steady-state condition.

Hill proposed an empirical function to describe the correlation between the active force generation, F, and the shortening velocity, V , as follows [29]:


(3.1) $$(F+a)(V+b)=(F_{max}+a)\cdot b,$$

where *a*, *b* and $\left(F_{max}+a\right)\cdot b=c$ are constants specific to each muscle type. The Hill model was first discovered on frog skeletal muscles. However, later studies have shown that the dimensionless form of this model with a shape factor $r=b/V_{max}=a/F_{max}\approx 0.25$ can be used to describe the behaviour of other muscle types,


(3.2) (f+r)(v+r)=(1+r)r,

where $f=\frac{F}{F_{max}}\;\text{and}\;v=\frac{V}{V_{max}}$ [88]. Hill also proposed a phenomenological model to describe muscle mechanics. The original model consists of a contractile element (CE) that generates force and an elastic element (SE), as shown in figure 2*a*. CE is governed by equation (3.2) and SE affects the length and rate of change in the length of CE during contractions [29,86]. Other forms of this model have also been introduced by integrating more elastic and viscous elements to account for the viscoelastic properties of the muscle and its interactions with the connective tissue surrounding muscle fibres [89]. The Hill model has enabled researchers to explore the mechanics of muscles using only a few rheological parameters. However, the Hill model falls short of elucidating the underlying biological mechanisms of force generation in muscles [89–91]. Another shortcoming of this model is that it fails to consider variations in the contractile characteristics of various fibre types within muscles and the dependence of muscle tension on the movement history [89,91]. Modifications aimed at enhancing the accuracy of the Hill-type model predictions are reviewed in [91].

This model is extremely versatile and not only can it be used to describe the behaviour of various types of muscles, but also it can be adapted and employed to describe force generation in non-muscle cells and their response to the physical and mechanical properties of their microenvironment [92–94]. Mitrossilis *et al*. [37] studied the response of a single C2.7 myoblast cell and a 3T3 fibroblast mounted between two microplates and showed a time-dependent force generation in single cells in response to the microplate stiffness (figure 2*b*(iii)). The force–velocity curves for single cells have a similar shape as the force–velocity curves reported for muscle fibres. Moreover, they showed that the reduced Hill equation (3.2), with the same shape factor r=0.25 as for muscles, can describe the normalized shortening speed, $v=V/V_{max}$ versus the normalized force, $f=F/F_{max}$ , as shown in figure 2*a*(ii). In these experiments, the maximum force and velocity are measured at infinite microplate stiffness and very low stiffnesses, respectively. Therefore, showing that the coupling between force generation in cells in contact with their surroundings still follows Hill’s model is quite remarkable, as it implies that structured muscle actomyosin and cytoskeletal actomyosin exhibit similar behaviours independent of the network architecture.

In addition, since the same function could explain the experimental results of different muscle types, Huxley suggested that the force–velocity relationship is generic in muscles and proposed a molecular explanation [95]. This molecular model incorporates the dynamics of the interaction between actin filaments and myosin motors, that is, the number of myosin heads connected to actin filaments and the formation of temporary connections between actin and myosin heads (i.e. cross bridges). Understanding these dynamics brings valuable insight into the biological processes regulating the predictions of the Hill model. For instance, cross-bridge-type models have shown that the maximum speed of muscle shortening happens owing to the rate of myosin attachment and detachment [89,95] rather than the extent of filament overlap [96,97]. This model has its limitations as well. For example, this model does not include the effect of power stroke (i.e. a crucial step in the force generation cycle in muscles where myosin heads pull the actin filaments and generate force) [89,91].

Further attempts have been made to incorporate various aspects of molecular biology with the Hill model. An example involves a model that integrated the dynamics of actin filament slippage during the process of force generation, allowing the prediction of the relationship between force generation and stiffness of micropillars [98]. This model was further refined to account for the temporal evolution of the force over time by integrating an internal variable, specifically representing the progression of myosin motor stalling over time [99]. Despite these improvements, this model falls short in explaining the limiting factors for the maximum force generation and maximum speed of shortening of the cells. A model that addresses these shortcomings is discussed in the next section.

### 3.2. Active element that includes the dynamic turnover of the actomyosin network

Étienne *et al*. developed an active fluid model to link the fundamental features of cellular molecular mechanics with a passive phenomenological model [38]. These features encompass the transient nature of the actomyosin network, force generation via myosin motor activity and actin polymerization. The transiently cross-linked actomyosin network is modelled as a Maxwell fluid in series with an active element $\sigma_{a}$ , mimicking force generation via myosin motors (figure 2*b*). The constitutive equation of the network is shown as follows:


(3.3) $$\tau_{\alpha }\overset{˙}{\sigma }+\sigma -2\tau_{\alpha }E\overset{˙}{\varepsilon }=\sigma_{a}.$$

Here, $\tau_{\alpha }$ is the characteristic time scale of cross-linker unbinding (i.e. elastic-like in short time scales ( $t<\tau_{\alpha }$ ) and viscous over longer periods of time ( $t>\tau_{\alpha }$ )), and E is the elastic modulus of the cell. $\sigma_{a}$ represents the maximum value of contractility or stall force that could be generated in cells, determined by two factors. First, it is influenced by the rate at which myosin motors can generate stress ( $1/\tau_{myo}$ ) and contract the cell with an elastic modulus of E . Second, it is affected by the rate of cross-linker unbinding ( $1/\tau_{\alpha }$ ), which counteracts the increase of stress in the system. Consequently, $\sigma_{a}$ is proportional to $(\tau_{\alpha }/\tau_{myo})E$ . This model effectively captures the evolution of force generation in cells leading to the establishment of tension in the steady state. Using a single dashpot (figure 2*b*(i)), instead of the proposed two (figure 2*b*(ii)), results in a similar constitutive equation. However, using two dashpots highlights the loss of force generation in the steady-state condition when the net displacement of the microplates is 0, indicating internal creep.

This model can predict the critical stiffness over which the plateau force ( $F_{p}$ ) remains constant, the response of cells to step changes in substrate stiffness, and the rate at which force is generated across various substrate stiffnesses. Interestingly, the constitutive equation (3.3) can also be written in a similar form as the Hill model of muscle contraction,


(3.4) $$\left(\frac{F}{S}+a\right)\left(v+b\right)=c,$$

where $a=E,b={2v}_{t}+v_{\alpha },$ and $c=\left(\sigma_{a}+E\right)v_{\alpha }-(L\overset{˙}{F})/(2S)$ . Here, $v=\dot{L}$ is the speed of shortening, $v_{t}$ represents the rate of actin polymerization and $v_{\alpha }=L/2\tau_{\alpha }$ is the internal creep. This equation allows the examination of non-muscle cell behaviour in two extreme cases: F=0 (stiffness of the substrate equal to 0) and v=0 (stiffness of the substrate equal to infinity). These extreme scenarios illustrate the role of the molecular mechanisms that both govern and limit cell responses. Basically, cells initiate force generation upon attachment to the substrate. If the resistance of the external environment is lower than the force generated via myosin motors (e.g. when k=0 ), cells will start to contract the microplate, which would, in turn, increase its resistance against the cell. This increased external resistance leads to a reduction in the rate of retrograde flow. Moreover, cross-linker unbinding and actin polymerization are two internal mechanisms that antagonize the rate of retrograde flow. This explanation can also be shown according to equation (3.4); when F is 0, the maximum shortening length is $v_{max}=\frac{\sigma_{a}L}{2\tau_{\alpha }E}-2v_{t}$ . These two internal factors also determine the maximum force generated in the system in the case of very high stiffnesses. As demonstrated by $F_{max}=\sigma_{a}S\left(1-\frac{E+\sigma_{a}}{\sigma_{a}}\frac{2v_{t}}{v_{\alpha }+2v_{t}}\right),$ the maximum force in the cell is not equal to $\sigma_{a}.$ In this scenario, actin polymerization requires extra work by myosin motors, which will be lost as a boundary creep. Furthermore, cross-linker unbinding will also result in force dissipation, leading to internal creep.

An analogous active Maxwell fluid model was employed to understand the mechanical characteristics underlying the observed cellular response to local RhoA activation [87]. In their experiments, Oakes *et al*. used optogenetic probes to recruit a cytosolic photo-recruitable protein RhoA-specific guanine exchange factor (prGEF) to the plasma membrane and activated RhoA over periods of 15 minutes. During local activation of RhoA, the fluorescent intensity of both actin and myosin II increased exponentially, plateaued and then decreased during the relaxation period (figure 2*c*(i)). A similar response has been observed while measuring traction forces and strain energy in cells (figure 2*c*(iii)). Besides, RhoA activation resulted in a sudden enhancement of traction force generation in the cell borders, whereas in the activation region traction forces did not change.

In the model used to describe this behaviour, contractile stress is introduced as an internal boundary condition, that is, $l_{0}<L$ , $\sigma \left(x=\pm l_{0},t\right)=\mp \sigma_{0}\left(t\right),$ to represent RhoA activation in the cell area. The profile of $\sigma_{0}$ follows a similar pattern as the actin and myosin accumulation in the activation area (figure 2*c*(ii)). In addition, a two-dimensional model of stress fibres embedded in a passive viscoelastic environment was used to estimate the direction and magnitude of the actomyosin flow towards the activation region. Finally, through the application of this model, they showcased the role of Zyxin in regulating the time scale of the initial elastic behaviour.

Active CEs are employed in chemomechanical models as well. These models integrate the effect of the interplay between the mechanical characteristics of cells, external and internal mechanical stimuli and the cascade of biochemical signals to simulate cell behaviour in various scenarios. For example, a chemomechanical model illustrated the growth dynamics of cell–ECM adhesion structures and highlighted the regulatory influence of the stiffness of the nucleus and ECM [100]. Another model demonstrated how the interaction between cells and ECM affects both gene expression and nuclear architecture [101]. This group of models is reviewed in [102,103].

## 4. Integrating activity in viscoelastic solid models

The previous section demonstrated how active tension originating from actomyosin dynamics can be integrated within constitutive equations that relate force and rate of contraction. Étienne *et al*.’s model provides, in particular, a detailed description of the transient regimes leading to the establishment of steady active stress [38]. These descriptions treat active materials as fluid without a reference to an intrinsic shape. It is, however, common for tissues to exhibit solid-like characteristics, with a well-defined reference shape, and the role of active stresses may then be interpreted as an apparent tension or a change in reference shape. In this section, we review these different approaches, highlighting their similarities and differences.

### 4.1. Constant active element

A simple way to introduce active contraction across a material is to include a stress-generating unit of constant value. Such an approach was used by Wyatt *et al*., when they investigated the short-time-scale response of Madin–Darby canine kidney (MDCK) monolayers to in-plane compressions [18], a process observed during morphogenetic processes [104,105] and the normal physiological function of many epithelial tissues [106,107]. The MDCK monolayers were placed between two rods and compressed at different rates. Quickly compressing the monolayer to strains below a threshold level (*ε* ~ 33%) resulted in transient folds that disappeared in time scales of the order of seconds, while the folds created owing to higher strains were permanent. The same buckling threshold of *ε* ~ 33% was observed when the monolayers were compressed both rapidly and at a low rate. In addition, using actomyosin inhibitors, they have demonstrated that actomyosin activity regulates the rate of tissue flattening, the buckling threshold, pretension and the long-time-scale stiffness of the monolayers.

**Figure 3.**
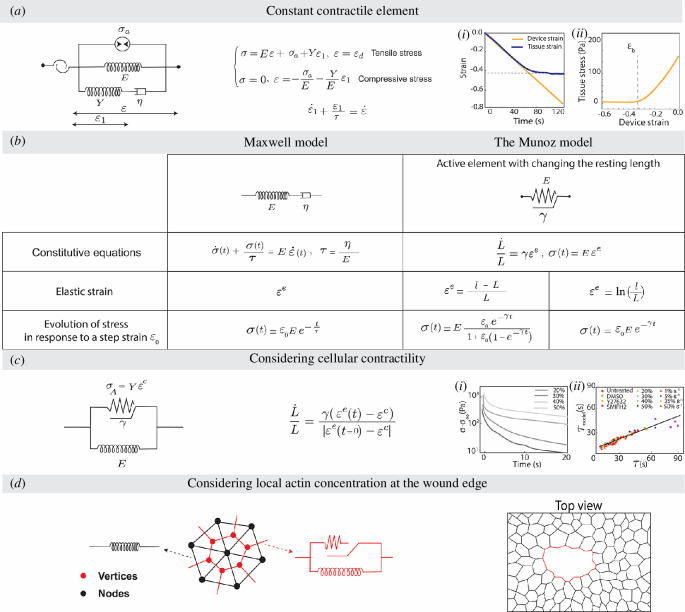
Active models for solid-like materials. (*a*). Active rheological model that includes a constant CE, $\sigma_{a}$ , in parallel to a standard solid model to account for the prestress in cell monolayers [18]. Temporal evolution of strain (**i**) and tissue stress (ii) as a function of device strain presented for epithelial monolayers undergoing compression at a low strain rate (0.5% $s^{-1}$ ). (*b*) The active element proposed by Muñoz *et al*., which adapts its resting length in response to deformations [108]. The evolution of stress over time in stress relaxation tests predicted by the Maxwell model and active models with two definitions of elastic strain is presented in a table. Stress in the models is defined using the elastic strain, that is, $\sigma \left(t\right)=E\varepsilon^{e}.$ Changing the definition of elastic strain can result in an equivalent evolution of stress in the active element and the Maxwell model for $\gamma \approx \tau^{-1}.$ When the strain is very small, the difference between the evolution of stresses in all three models will be negligible. However, at larger strains, the stress in the system would be dependent on the way that the strain is defined. (*c*) The modified version of the active element proposed by Muñoz *et al.*, which also considers the effect of cellular contractility [109]. (**i**) The average curves representing the evolution of stress over time for MDCK monolayers stretched at 75% $s^{-1}$ to various amplitudes of strain plotted on a semi-log scale. (ii) Demonstration of the correlation between the $\tau_{\text{model}}$ calculated from the active rheological model and the characteristic time τ calculated from fitting the stress–time curves with an empirical function, that is, $\sigma =Ae^{-t/\tau }t^{-\alpha }+B$ , for different loading conditions and actomyosin treatments. (*d*) An example of a hybrid vertex model employed to study wound healing. The top view of the epithelial tissue with ablated cells is shown along with the rheological models of nodal segments (cell-centre connections) in black and vertex segments (cell boundaries) in red [110].


A simple active rheological model could reproduce the results of their studies conducted under different loading conditions (figure 3*a*). The model consists of a constant active element $\sigma_{a}$ , which brings the system to a tensile state even at 0 external load, in parallel to a standard linear solid model. The MDCK monolayer buckles when the stress in the monolayer approaches the compression range. Therefore, under compressive strains that would normally cause compressive stress, the model assumes that stress in the monolayer remains 0. The constitutive equation under tensile and compressive stresses is defined as

(4.1) $$\left\{ \begin{array}{llll} \sigma =E\varepsilon +\sigma_{a}+Y\varepsilon_{1} & \text{and} & \varepsilon =\varepsilon_{d} & \text{under tensile stress,}\\ \sigma =0 & \text{and} & \varepsilon =\frac{-\sigma_{a}}{E}-\frac{Y}{E}\varepsilon_{1} & \text{under compressive stress,} \end{array}\right.$$

where $\sigma_{a}$ is the pretension in the monolayer, $\varepsilon_{d}$ is the device strain, E characterizes the long-term stiffness of the tissue, Y and η describe the short-time-scale response, $\varepsilon_{1}$ is the strain in the spring with stiffness Y satisfying ${\dot{\varepsilon }}_{1}+\frac{\varepsilon_{1}}{\tau }=\dot{\varepsilon }$ , and τ=η/Y is the characteristic time scale. Based on the model predictions for the steady state, when the applied strain is larger than the buckling threshold $\varepsilon_{b}$ , $\varepsilon >\varepsilon_{b}$ , tissue strain will be equal to the device strain, $\varepsilon =\varepsilon_{d}$ and the stress in the tissue will follow $\sigma =E\varepsilon_{d}+\sigma_{a}$ . However, when the monolayer is compressed past the model buckling threshold, stress levels fall to 0 and tissue strain plateaus at $\varepsilon_{b}=-\sigma_{a}/E$ . These model predictions are consistent with the experimental results shown in figure 3*a*(i) and (ii). The model can also predict the response of the monolayer to a step of compressive strain of different magnitudes. Besides, the model provides a simple way to capture how treatments affecting actomyosin control the amount of active tension revealing that the buckling threshold observed in the experiments agrees with model predictions $-\sigma_{a}/E$ in all conditions.

### 4.2. Active behaviour modelled as change in the resting length of a spring

The previous approach focused on how active stress, combined with external perturbations, would set the mechanical evolution of a viscoelastic tissue. The Maxwell branch and its dashpot, in particular, account for the remodelling and plasticity of the material. Another way to represent this is through the explicit evolution of the rest shape, or stress-free state, of the material. Both remodelling and tensioning can be accounted for through the evolution of the resting length of cells and tissues over time, as proposed by Muñoz *et al*. [108]. We first describe how a Maxwell-like behaviour can emerge from this strategy and then how activity can be added to this class of models.

#### 4.2.1. Model proposed by Muñoz *et al.*

Muñoz *et al*. proposed a model to account for the influence of cytoskeleton activity on cell shape by representing plasticity as a change in the rest length of a spring [108]. A material’s constitutive equation therefore takes the form of a relationship between the rate of change of the rest length and the stress (or equivalently some metric of elastic strain) in the material. In this model, the current resting length of the material (filament, cell or tissue) L , that is, the total length of the material when no external load is applied to it, is proportional to the elastic strain $\varepsilon^{e}$ . The rate of changing the resting length under strain is defined as


(4.2) $$\frac{\overset{˙}{L}}{L}=\gamma \varepsilon^{e},$$

where γ is the remodelling rate of the network defined as the network resistance to adjusting its configuration to the applied deformation, $\varepsilon^{e}$ is the current elastic strain, $\varepsilon^{e}=\frac{l-L}{L}$ and l is the current total length of the network. According to Muñoz’s model, the definition of current elastic strain is different from the apparent strain $\varepsilon =\frac{l-L_{0}}{L_{0}}$ , where $L_{0}$ is the initial length and the resting length of the network.

For small deformations, Muñoz’s approach is mathematically equivalent to a linear Maxwell model with a characteristic time of $\tau =\frac{\eta }{E}$ [108]. However, at large deformation, the models differ, leading to rather complex relaxation dynamics for Muñoz’s model (see figure 3*b*). This difference results from the particular definitions of strains and the resulting nonlinearities emerging from them. For instance, using a logarithmic form for the elastic strain $\varepsilon^{e}=\ln\left(\frac{l}{L}\right)$ (i.e. using the true strain definition rather than the engineering strain), Muñoz’s model would match the Maxwell model up to large deformations, with a relaxation time scale independent of the strain amplitude, as shown in the table in figure 3*b*. At a mathematical level, controlling the rest length of a spring or having a dashpot in series is therefore largely equivalent.

However, introducing a dynamic rest length enables a slightly different interpretation of the physiological mechanisms [108]. The dashpot element in the Maxwell model is often associated with remodelling the system but could also account for the resistance of the cytoplasmic fluid η to the applied strain rate and dissipate power. On the other hand, for Muñoz’s active element, the inelastic part of the external power will be used to overcome the resistance of the cytoskeleton filaments γ to adapt to the new configuration imposed by the external strain. The active model proposed by Muñoz has been generalized to two-dimensional/three-dimensional continuum models [111] and also integrated into discrete models such as cell-centred [112], vertex [113] and cell-centred/vertex hybrid [109,110] approaches. By incorporating a porosity parameter representing the density of polymers in the cell cytoskeleton, the continuum model proposed by Asadipour *et al*. can also replicate the immediate fluidization in cells in response to transient strains and the subsequent gradual stiffening [111]. These adaptations have facilitated the study of epithelial tissue behaviour in both two and three dimensions. A modelling approach similar to the Muñoz model proposed by Esfahani *et al*. could also demonstrate stiffening in response to high strain rates applied to epithelial cell pairs [49]. A few of the modifications made to the Muñoz model are presented in the following sections.

#### 4.2.2. Active element considering cellular contractility

Mosaffa *et al*. [109] modified the evolution law of the resting length of the material proposed by Muñoz *et al*. [108] by introducing a contractility parameter $\varepsilon^{c}$ to account for the inherent contractility of the cells,


(4.3) $$\frac{\overset{˙}{L}}{L}=\gamma \left(\varepsilon^{e}-\varepsilon^{c}\right).$$

In this model, when the elastic strain $\varepsilon^{e}$ reaches to $\varepsilon^{c}$ , the resting length will not change any more, and as previously stated [108], when $\varepsilon^{c}$ is zero, the model behaves similarly to the Maxwell model. Mosaffa *et al*. implemented the modified active element in a hybrid cell-centred/vertex model where cells interact through both cell centrs, presented by nodes and cell–cell junctions, presented by the connection between vertices [109]. This model could successfully simulate tissue extension and wound healing.

Khalilgharibi *et al*. have used a similar approach for fitting the results of stress relaxation tests conducted on MDCK monolayers [5]. Their studies have shown that stress in the MDCK monolayer increases promptly after strain application. Then, the stress will gradually relax along with an increase in the monolayer length, which is regulated by actomyosin activity. Moreover, they have noticed a strain-dependent characteristic time τ for monolayers stretched at 75% $s^{-1}$ strain rate (figure 3*c*(i)), which cannot be explained by standard linear viscoelastic models. Therefore, they proposed a model that consists of an elastic spring in parallel to an active element that sustains a constant pre-strain $\varepsilon^{c}$ and changes its resting length $L\left(t\right)$ to relax the imposed stress and return its strain to $\varepsilon^{c}$ . The changing of the resting length of the monolayer L(t) in response to an applied strain $\varepsilon_{0}$ is defined as


(4.4) $$\frac{\overset{˙}{L}}{L}=\frac{\gamma \left(\varepsilon^{e}\left(t\right)-\varepsilon^{c}\right)}{\left|\varepsilon^{e}\left(t=0s\right)-\varepsilon^{c}\right|}.$$

Here, $\varepsilon^{e}(t)$ is the effective strain $\varepsilon^{e}\left(t\right)=\left(l_{m}-L\left(t\right)\right)/L\left(t\right)$ with $l_{m}$ representing the actual length of the monolayer after applying the deformation and γ is the rate of changing the resting length. The characteristic time predicted by this model, $\tau_{model}=\varepsilon_{0}/[\gamma (1+\varepsilon_{0})]$ , increases with the applied strain, which is consistent with their experimental observations, as shown in (figure 3*c*(ii)).

Another example of the active element that accounts for cellular contractility involves integrating this model into a hybrid two-dimensional cell-centred/vertex model to analyse the wound healing process [110]. In this model, the vertex segments (cell boundaries) and nodal segments (cell-centre connections) connect the apical and basal sides. Nodal segments are characterized using an elastic spring, and the behaviour of vertex segments is described by an active model consisting of two branches in parallel (figure 3*d*). The first branch is an active element that accounts for the changes in the resting length of the vertex following equation (4.3). The second branch is an elastic spring with an additional time-varying contractility parameter ${\hat{\Upsilon }}^{c},$ which accounts for the effect of high actin concentration at the wound edge (i.e. purse string contractility) and increases the stress in the elastic spring. The outcomes of their simulations have demonstrated the regulatory mechanism of both purse string contractility and tissue contractility on wound healing speed.

### 4.3. Active element considering a time delay in the active rest length changes

One of the potential reasons for the oscillatory response observed in tissues during various processes, such as morphogenesis, could be the delay between the signal and the response, as stated by Muñoz *et al*. [113]. These delays in the responses can be owing to the distance between the sender and receiver of the biochemical signals or the time necessary for signal processing [113,114]. To study this phenomenon, they modified the active element that was previously proposed by Muñoz *et al*. by considering the effect of a time delay between the mechanical signals and the active rest length changes [108],


(4.5) $$\overset{˙}{L}=\gamma \left[l\left(t-\tau \right)-L\left(t-\tau \right)\right].$$

Analysing the stability of the delay differential equation resulted in the limits of oscillation and stability as follows:


(4.6) $$\tau_{oscil}=\frac{1}{e\gamma }\;and\;\tau_{stab}=\frac{\pi }{2\gamma }.$$

$\tau_{\text{oscil}}$ is the time beyond which the rest length of the element oscillates, e is the exponential constant, and for time scales above $\tau_{\text{stab}}$ the value of the rest length is unstable, and its oscillation amplitude will rise over time. These values might trigger oscillations during embryogenesis.

In addition, delays can also be dependent on the apparent size of the element $l\left(t\right).$ Muñoz *et al*. implemented the effect of the size-dependent delays, $\tau \left(t\right)=\lambda l\left(t\right)$ , into a vertex model to analyse the oscillation in the cellular area in biological tissues [113]. In the vertex model, the rest length of the nodal elements was maintained constant, and changes in the resting length of the vertex elements were defined using equation (4.5). For constant delays, oscillations in the cell area were periodic and synchronous. However, for size-dependent delays, oscillations started to get increasingly out of sync. It is argued that this model demonstrated the role of delay in the mechanical response in inducing oscillations even in the absence of external sources.

## 5. Summary and future perspectives

The active viscoelastic models outlined here are capable of capturing numerous aspects of cell behaviour at multiple spatial and temporal scales with a small number of model parameters without considering the details of the structural components and dynamics of cell–cell and cell–ECM adhesion sites. These active models provided insight into the results of experiments and predicted the system behaviour in other arbitrary conditions. Additionally, despite the difficulty in establishing a clear connection between biological processes and model parameters, researchers have used drug treatments or targeted mutations to demonstrate correlations between model parameters like Young’s modulus, viscosity or active pretension and biological processes such as actomyosin activity, even molecules that regulate these processes.

Each active viscoelastic model is described by a constitutive equation that represents a particular cellular behaviour, such as sensing changes in the substrate stiffness, and response to strains at different magnitudes and rates in cell doublets and cell monolayers. Therefore, the existence and use of a generalized model that can be employed to describe and predict the response of cells in different scenarios is still an open question. Integrating and bridging the gap between phenomenological and biophysical models is an important step to improve our understanding of these systems. For example, a molecular model of the actomyosin cortex inspired a phenomenological model for cell-scale mechanosensing [38], and a phenomenological model that included the role of actin polymerization in changing the resting length of the material was incorporated in vertex models to study different aspects of tissue dynamics [113]. Consequently, a comprehensive phenomenological model that captures all the significant facets of rheological data might also enrich the findings of biophysical models and allow us to improve the precision of simulations of cell activity.

Numerical models are an excellent research tool to complement, analyse and interpret experimental data in the field of cell mechanics. Numerous models with varying degrees of complexity and details of the structural elements involved in the observed phenomena have been presented over the years. Power law [15], fractional (reviewed in [17]), viscoelastic and active viscoelastic models are included in this category, where the effects of subcellular microstructures on cell rheology are represented by model parameters. For example, virtual cell (VCell) is a powerful model that includes details of the nucleus, cytoskeleton, cytoplasm and chromatin fibres [115]. This level of detail is computationally expensive and might not always be necessary. In other words, based on the research question, the phenomenon of interest, length and time scales and characteristics of the relevant microstructures can be incorporated into biophysical models. For example, at the molecular scale, chemomechanical [103,116] and molecular clutch models [117–119] are used to study cell–cell and cell–ECM adhesions, at the cell-scale statistical approaches can be employed to study cell mechanics [120–122], and cellular Potts (CPM) [123–125], vertex [126,127] and self-propelled Voronoi [128–130] models are introduced to study the mechanical behaviour of epithelial monolayers in two dimensions and three dimensions. The level of detail can be further reduced by describing the outcomes of experiments using phenomenological models before examining the underlying mechanisms.

## Data Availability

This article has no additional data.

## References

[1] Fritsch A , Höckel M , Kiessling T , Nnetu KD , Wetzel F , Zink M , Käs JA . 2010 Are biomechanical changes necessary for tumour progression? Nat. Phys. **6** , 730–732. (10.1038/nphys1800)

[2] Phipps S , Yang THJ , Habib FK , Reuben RL , McNeill SA . 2005 Measurement of tissue mechanical characteristics to distinguish between benign and malignant prostatic disease. Urology **66** , 447–450. (10.1016/j.urology.2005.03.017) 16098374

[3] Palacio-Torralba J , Hammer S , Good DW , Alan McNeill S , Stewart GD , Reuben RL , Chen Y . 2015 Quantitative diagnostics of soft tissue through viscoelastic characterization using time-based instrumented palpation. J. Mech. Behav. Biomed. Mater. **41** , 149–160. (10.1016/j.jmbbm.2014.09.027) 25460411

[4] Khalilgharibi N , Fouchard J , Recho P , Charras G , Kabla A . 2016 The dynamic mechanical properties of cellularised aggregates. Curr. Opin. Cell Biol. **42** , 113–120. (10.1016/j.ceb.2016.06.003) 27371889

[5] Khalilgharibi N *et al* . 2019 Stress relaxation in epithelial monolayers is controlled by the actomyosin cortex. Nat. Phys. **15** , 839–847. (10.1038/s41567-019-0516-6) 33569083 PMC7116713

[6] Forgacs G , Foty RA , Shafrir Y , Steinberg MS . 1998 Viscoelastic properties of living embryonic tissues: a quantitative study. Biophys. J. **74** , 2227–2234. (10.1016/S0006-3495(98)77932-9) 9591650 PMC1299566

[7] Guevorkian K , Colbert MJ , Durth M , Dufour S , Brochard-Wyart F . 2010 Aspiration of biological viscoelastic drops. Phys. Rev. Lett. **104** , 218101. (10.1103/PhysRevLett.104.218101) 20867138

[8] Charras G , Yap AS . 2018 Tensile forces and mechanotransduction at cell-cell junctions. Curr. Biol. **28** , R445–R457. (10.1016/j.cub.2018.02.003) 29689229

[9] Prost J , Jülicher F , Joanny JF . 2015 Active gel physics. Nature Phys. **11** , 111–117. (10.1038/nphys3224)

[10] Aigouy B , Farhadifar R , Staple DB , Sagner A , Röper JC , Jülicher F , Eaton S . 2010 Cell flow reorients the axis of planar polarity in the wing epithelium of Drosophila. Cell **142** , 773–786. (10.1016/j.cell.2010.07.042) 20813263

[11] Wyatt TPJ , Harris AR , Lam M , Cheng Q , Bellis J , Dimitracopoulos A , Kabla AJ , Charras GT , Baum B . 2015 Emergence of homeostatic epithelial packing and stress dissipation through divisions oriented along the long cell axis. Proc. Natl Acad. Sci. USA **112** , 5726–5731. (10.1073/pnas.1420585112) 25908119 PMC4426437

[12] Hart KC , Tan J , Siemers KA , Sim JY , Pruitt BL , Nelson WJ , Gloerich M . 2017 E-cadherin and LGN align epithelial cell divisions with tissue tension independently of cell shape. Proc. Natl Acad. Sci. **114** , E5845–E5853. (10.1073/pnas.1701703114) 28674014 PMC5530667

[13] Trepat X , Deng L , An SS , Navajas D , Tschumperlin DJ , Gerthoffer WT , Butler JP , Fredberg JJ . 2007 Universal physical responses to stretch in the living cell. Nature **447** , 592–595. (10.1038/nature05824) 17538621 PMC2440511

[14] Krishnan R *et al* . 2009 Reinforcement versus fluidization in cytoskeletal mechanoresponsiveness. PLoS one **4** , e5486. (10.1371/journal.pone.0005486) 19424501 PMC2675060

[15] Kollmannsberger P , Fabry B . 2011 Linear and nonlinear rheology of living cells. Annu. Rev. Mater. Res. **41** , 75–97. (10.1146/annurev-matsci-062910-100351)

[16] Rosowski KA , Boltyanskiy R , Xiang Y , Van den Dries K , Schwartz MA , Dufresne ER . 2018 Vinculin and the mechanical response of adherent fibroblasts to matrix deformation. Sci. Rep. **8** , 17967. (10.1038/s41598-018-36272-9) 30568231 PMC6299284

[17] Bonfanti A , Kaplan JL , Charras G , Kabla A . 2020 Fractional viscoelastic models for power-law materials. Soft Matter **16** , 6002–6020. (10.1039/d0sm00354a) 32638812

[18] Wyatt TPJ , Fouchard J , Lisica A , Khalilgharibi N , Baum B , Recho P , Kabla AJ , Charras GT . 2020 Actomyosin controls planarity and folding of epithelia in response to compression. Nat. Mater. **19** , 109–117. (10.1038/s41563-019-0461-x) 31451778

[19] Darling EM , Di Carlo D . 2015 High-throughput assessment of cellular mechanical properties. Annu. Rev. Biomed. Eng. **17** , 35–62. (10.1146/annurev-bioeng-071114-040545) 26194428 PMC8204286

[20] Quan FS , Kim KS . 2016 Medical applications of the intrinsic mechanical properties of single cells. Acta Biochim. Biophys. Sin. **48** , 865–871. (10.1093/abbs/gmw081) 27542404

[21] Cross SE , Jin YS , Rao J , Gimzewski JK . 2007 Nanomechanical analysis of cells from cancer patients. Nat. Nanotechnol. **2** , 780–783. (10.1038/nnano.2007.388) 18654431

[22] Wong VW , Akaishi S , Longaker MT , Gurtner GC . 2011 Pushing back: wound mechanotransduction in repair and regeneration. J. Invest. Dermatol. **131** , 2186–2196. (10.1038/jid.2011.212) 21776006

[23] Wozniak MA , Chen CS . 2009 Mechanotransduction in development: a growing role for contractility. Nat. Rev. Mol. Cell Biol. **10** , 34–43. (10.1038/nrm2592) 19197330 PMC2952188

[24] Balasubramaniam L , Mège RM , Ladoux B . 2022 Active nematics across scales from cytoskeleton organization to tissue morphogenesis. Curr. Opin. Genet. Dev. **73** , 101897. (10.1016/j.gde.2021.101897) 35063879

[25] Doostmohammadi A , Ignés-Mullol J , Yeomans JM , Sagués F . 2018 Active nematics. Nat. Commun. **9** , 3246. (10.1038/s41467-018-05666-8) 30131558 PMC6104062

[26] Needleman D , Dogic Z . 2017 Active matter at the interface between materials science and cell biology. Nat. Rev. Mater. **2** , 1–14. (10.1038/natrevmats.2017.48)

[27] Kumar KV . 2021 The actomyosin cortex of cells: a thin film of active matter. J. Indian Inst. Sci. **101** , 97–112. (10.1007/s41745-020-00220-2)

[28] Brown AL , Farhat W , Merguerian PA , Wilson GJ , Khoury AE , Woodhouse KA . 2002 22 week assessment of bladder acellular matrix as a bladder augmentation material in a porcine model. Biomaterials **23** , 2179–2190. (10.1016/s0142-9612(01)00350-7) 11962659

[29] Hill AV . 1938 The heat of shortening and the dynamic constants of muscle. Proc. R. Soc. Lond. B **126** , 136–195. (10.1098/rspb.1938.0050)

[30] Harris AR , Bellis J , Khalilgharibi N , Wyatt T , Baum B , Kabla AJ , Charras GT . 2013 Generating suspended cell monolayers for mechanobiological studies. Nat. Protoc. **8** , 2516–2530. (10.1038/nprot.2013.151) 24263091

[31] Crichton ML , Donose BC , Chen X , Raphael AP , Huang H , Kendall MAF . 2011 The viscoelastic, hyperelastic and scale dependent behaviour of freshly excised individual skin layers. Biomaterials **32** , 4670–4681. (10.1016/j.biomaterials.2011.03.012) 21458062

[32] Uriarte JJ , Meirelles T , Gorbenko Del Blanco D , Nonaka PN , Campillo N , Sarri E , Navajas D , Egea G , Farré R . 2016 Early impairment of lung mechanics in a murine model of Marfan syndrome. PLoS one **11** , e0152124. (10.1371/journal.pone.0152124) 27003297 PMC4803219

[33] Okajima T . 2012 Atomic force microscopy for the examination of single cell rheology. Curr. Pharm. Biotechnol. **13** , 2623–2631. (10.2174/138920101314151120122846) 22039813

[34] Weafer PP , Reynolds NH , Jarvis SP , McGarry JP . 2015 Single cell active force generation under dynamic loading – Part I: AFM experiments. Acta Biomater. **27** , 236–250. (10.1016/j.actbio.2015.09.006) 26360596

[35] Fischer-Friedrich E , Toyoda Y , Cattin CJ , Müller DJ , Hyman AA , Jülicher F . 2016 Rheology of the active cell cortex in mitosis. Biophys. J. **111** , 589–600. (10.1016/j.bpj.2016.06.008) 27508442 PMC4982928

[36] Helenius J , Heisenberg CP , Gaub HE , Muller DJ . 2008 Single-cell force spectroscopy. J. Cell Sci. **121** , 1785–1791. (10.1242/jcs.030999) 18492792

[37] Mitrossilis D , Fouchard J , Guiroy A , Desprat N , Rodriguez N , Fabry B , Asnacios A . 2009 Single-cell response to stiffness exhibits muscle-like behavior. Proc. Natl Acad. Sci. USA **106** , 18 243–18 248. (10.1073/pnas.0903994106) 19805036 PMC2775285

[38] Étienne J , Fouchard J , Mitrossilis D , Bufi N , Durand-Smet P , Asnacios A . 2015 Cells as liquid motors: mechanosensitivity emerges from collective dynamics of actomyosin cortex. Proc. Natl Acad. Sci. USA **112** , 2740–2745. (10.1073/pnas.1417113112) 25730854 PMC4352826

[39] Bonakdar N , Gerum R , Kuhn M , Spörrer M , Lippert A , Schneider W , Aifantis KE , Fabry B . 2016 Mechanical plasticity of cells. Nat. Mater. **15** , 1090–1094. (10.1038/nmat4689) 27376682

[40] Kilinc D , Lee GU . 2014 Advances in magnetic tweezers for single molecule and cell biophysics. Integr. Biol. **6** , 27–34. (10.1039/c3ib40185e) 24263142

[41] Ayala YA *et al* . 2016 Rheological properties of cells measured by optical tweezers. BMC Biophys. **9** , 5. (10.1186/s13628-016-0031-4) 27340552 PMC4917937

[42] Arbore C , Perego L , Sergides M , Capitanio M . 2019 Probing force in living cells with optical tweezers: from single-molecule mechanics to cell mechanotransduction. Biophys. Rev. **11** , 765–782. (10.1007/s12551-019-00599-y) 31612379 PMC6815294

[43] Nussenzveig HM . 2018 Cell membrane biophysics with optical tweezers. Eur. Biophys. J. **47** , 499–514. (10.1007/s00249-017-1268-9) 29164289

[44] Hochmuth RM . 2000 Micropipette aspiration of living cells. J. Biomech. **33** , 15–22. (10.1016/s0021-9290(99)00175-x) 10609514

[45] González-Bermúdez B , Guinea GV , Plaza GR . 2019 Advances in micropipette aspiration: applications in cell biomechanics, models, and extended studies. Biophys. J. **116** , 587–594. (10.1016/j.bpj.2019.01.004) 30683304 PMC6383002

[46] Wang H , Zhou F , Guo Y , Ju LA . 2022 Micropipette-based biomechanical nanotools on living cells. Eur. Biophys. J. **51** , 119–133. (10.1007/s00249-021-01587-5) 35171346 PMC8964576

[47] Vedula SRK , Lim TS , Kausalya PJ , Lane EB , Rajagopal G , Hunziker W , Lim CT . 2009 Quantifying forces mediated by integral tight junction proteins in cell–cell adhesion. Exp. Mech. **49** , 3–9. (10.1007/s11340-007-9113-1)

[48] Tashiro H , Uchida M , Sato-Maeda M . 1993 Three-dimensional cell manipulator by means of optical trapping for the specification of cell-to-cell adhesion. Opt. Eng. **32** , 2812. (10.1117/12.147714)

[49] Esfahani AM *et al* . 2021 Characterization of the strain-rate-dependent mechanical response of single cell-cell junctions. Proc. Natl Acad. Sci. USA **118** , e2019347118. (10.1073/pnas.2019347118) 33531347 PMC7896335

[50] Yang R , Broussard JA , Green KJ , Espinosa HD . 2018 Techniques to stimulate and interrogate cell-cell adhesion mechanics. Extreme Mech. Lett. **20** , 125–139. (10.1016/j.eml.2017.12.002) 30320194 PMC6181239

[51] Monemian Esfahani A , Rosenbohm J , Reddy K , Jin X , Bouzid T , Riehl B , Kim E , Lim JY , Yang R . 2019 Tissue regeneration from mechanical stretching of cell-cell adhesion. Tissue Eng. Part C. Methods **25** , 631–640. (10.1089/ten.TEC.2019.0098) 31407627 PMC6859692

[52] Dahl KN , Engler AJ , Pajerowski JD , Discher DE . 2005 Power-law rheology of isolated nuclei with deformation mapping of nuclear substructures. Biophys. J. **89** , 2855–2864. (10.1529/biophysj.105.062554) 16055543 PMC1366783

[53] Davidson PM , Fedorchak GR , Mondésert-Deveraux S , Bell ES , Isermann P , Aubry D , Allena R , Lammerding J . 2019 High-throughput microfluidic micropipette aspiration device to probe time-scale dependent nuclear mechanics in intact cells. Lab Chip **19** , 3652–3663. (10.1039/c9lc00444k) 31559980 PMC6810812

[54] Hobson CM , Falvo MR , Superfine R . 2021 A survey of physical methods for studying nuclear mechanics and mechanobiology. APL Bioeng. **5** , 041508. (10.1063/5.0068126) 34849443 PMC8604565

[55] Tlili S , Gay C , Graner F , Marcq P , Molino F , Saramito P . 2015 Colloquium: mechanical formalisms for tissue dynamics. Eur. Phys. J. E. **38** , 1–31. (10.1140/epje/i2015-15033-4) 25957180

[56] Bernheim-Groswasser A , Gov NS , Safran SA , Tzlil S . 2018 Living matter: mesoscopic active materials. Adv. Mater. **30** , e1707028. (10.1002/adma.201707028) 30256463

[57] Banerjee S , Gardel ML , Schwarz US . 2020 The actin cytoskeleton as an active adaptive material. Annu. Rev. Condens. Matter Phys. **11** , 421–439. (10.1146/annurev-conmatphys-031218-013231) 33343823 PMC7748259

[58] Fletcher DA , Mullins RD . 2010 Cell mechanics and the cytoskeleton. Nature **463** , 485–492. (10.1038/nature08908) 20110992 PMC2851742

[59] Xi W , Saw TB , Delacour D , Lim CT , Ladoux B . 2019 Material approaches to active tissue mechanics. Nat. Rev. Mater. **4** , 23–44. (10.1038/s41578-018-0066-z)

[60] Carlier MF , Shekhar S . 2017 Global treadmilling coordinates actin turnover and controls the size of actin networks. Nat. Rev. Mol. Cell Biol. **18** , 389–401. (10.1038/nrm.2016.172) 28248322

[61] Pollard TD , Berro J . 2009 Mathematical models and simulations of cellular processes based on actin filaments. J. Biol. Chem. **284** , 5433–5437. (10.1074/jbc.R800043200) 18940808

[62] Pollard TD . 2016 Theory from the Oster laboratory leaps ahead of experiment in understanding actin-based cellular motility. Biophys. J. **111** , 1589–1592. (10.1016/j.bpj.2016.08.044) 27760345 PMC5071581

[63] Wegner A . 1976 Head to tail polymerization of actin. J. Mol. Biol. **108** , 139–150. (10.1016/s0022-2836(76)80100-3) 1003481

[64] Banerjee S , Gardel ML , Schwarz US . 2020 The actin cytoskeleton as an active adaptive material. Annu. Rev. Condens. Matter Phys. **11** , 421–439. (10.1146/annurev-conmatphys-031218-013231) 33343823 PMC7748259

[65] Neuhaus JM , Wanger M , Keiser T , Wegner A . 1983 Treadmilling of actin. J. Muscle Res. Cell Motil. **4** , 507–527. (10.1007/BF00712112) 6358256

[66] Chaudhuri O , Cooper-White J , Janmey PA , Mooney DJ , Shenoy VB . 2020 Effects of extracellular matrix viscoelasticity on cellular behaviour. Nature **584** , 535–546. (10.1038/s41586-020-2612-2) 32848221 PMC7676152

[67] Alert R , Trepat X . 2020 Physical models of collective cell migration. Annu. Rev. Condens. Matter Phys **11** , 77–101. (10.1146/annurev-conmatphys-031218-013516)

[68] Robert-Paganin J , Pylypenko O , Kikuti C , Sweeney HL , Houdusse A . 2020 Force generation by myosin motors: a structural perspective. Chem. Rev. **120** , 5–35. (10.1021/acs.chemrev.9b00264) 31689091

[69] Houdusse A , Sweeney HL . 2016 How myosin generates force on actin filaments. Trends Biochem. Sci. **41** , 989–997. (10.1016/j.tibs.2016.09.006) 27717739 PMC5123969

[70] Murrell M , Oakes PW , Lenz M , Gardel ML . 2015 Forcing cells into shape: the mechanics of actomyosin contractility. Nat. Rev. Mol. Cell Biol. **16** , 486–498. (10.1038/nrm4012) 26130009 PMC7443980

[71] Livne A , Geiger B . 2016 The inner workings of stress fibers − from contractile machinery to focal adhesions and back. J. Cell Sci. **129** , 1293–1304. (10.1242/jcs.180927) 27037413

[72] Yap AS , Duszyc K , Viasnoff V . 2018 Mechanosensing and mechanotransduction at cell-cell junctions. Cold Spring Harb. Perspect. Biol. **10** , a028761. (10.1101/cshperspect.a028761) 28778874 PMC6071489

[73] Shukla VC , Higuita-Castro N , Nana-Sinkam P , Ghadiali SN . 2016 Substrate stiffness modulates lung cancer cell migration but not epithelial to mesenchymal transition. J. Biomed. Mater. Res. A. **104** , 1182–1193. (10.1002/jbm.a.35655) 26779779

[74] Hadden WJ *et al* . 2017 Stem cell migration and mechanotransduction on linear stiffness gradient hydrogels. Proc. Natl Acad. Sci. USA **114** , 5647–5652. (10.1073/pnas.1618239114) 28507138 PMC5465928

[75] Alert R , Trepat X . 2019 Physical models of collective cell migration. arXiv. See https://arxiv.org/abs/1905.07675

[76] Sun M et al . 2018 Effects of matrix stiffness on the morphology, adhesion, proliferation and osteogenic differentiation of mesenchymal stem cells. Int. J. Med. Sci. **15** , 257–268. (10.7150/ijms.21620) 29483817 PMC5820855

[77] Liu N et al . 2018 Effect of substrate stiffness on proliferation and differentiation of periodontal ligament stem cells. Cell Prolif. **51** , e12478. (10.1111/cpr.12478) 30039894 PMC6528973

[78] Engler AJ , Sen S , Sweeney HL , Discher DE . 2006 Matrix elasticity directs stem cell lineage specification. Cell **126** , 677–689. (10.1016/j.cell.2006.06.044) 16923388

[79] Mao AS , Shin JW , Mooney DJ . 2016 Effects of substrate stiffness and cell-cell contact on mesenchymal stem cell differentiation. Biomaterials **98** , 184–191. (10.1016/j.biomaterials.2016.05.004) 27203745 PMC4906313

[80] Li B , Wang JHC . 2011 Fibroblasts and myofibroblasts in wound healing: force generation and measurement. J. Tissue Viability **20** , 108–120. (10.1016/j.jtv.2009.11.004) 19995679 PMC2891362

[81] Bosveld F *et al* . 2012 Mechanical control of morphogenesis by Fat/Dachsous/Four-jointed planar cell polarity pathway. Science **336** , 724–727. (10.1126/science.1221071) 22499807

[82] Yamada KM *et al* . 2019 Extracellular matrix dynamics in cell migration, invasion and tissue morphogenesis. Int. J. Exp. Pathol. **100** , 144–152. (10.1111/iep.12329) 31179622 PMC6658910

[83] Chowdhury F , Huang B , Wang N . 2021 Cytoskeletal prestress: the cellular hallmark in mechanobiology and mechanomedicine. Cytoskeleton (Hoboken) **78** , 249–276. (10.1002/cm.21658) 33754478 PMC8518377

[84] Rodriguez ML , McGarry PJ , Sniadecki NJ . 2013 Review on cell mechanics: experimental and modeling approaches. Appl. Mech. Rev. **65** . (10.1115/1.4025355)

[85] Doss BL , Pan M , Gupta M , Grenci G , Mège RM , Lim CT , Sheetz MP , Voituriez R , Ladoux B . 2020 Cell response to substrate rigidity is regulated by active and passive cytoskeletal stress. Proc. Natl Acad. Sci. USA **117** , 12 817–12 825. (10.1073/pnas.1917555117) 32444491 PMC7293595

[86] Herzog W . 2019 The problem with skeletal muscle series elasticity. BMC Biomed. Eng. **1** , 28. (10.1186/s42490-019-0031-y) 32903293 PMC7422574

[87] Oakes PW , Wagner E , Brand CA , Probst D , Linke M , Schwarz US , Glotzer M , Gardel ML . 2017 Optogenetic control of RhoA reveals zyxin-mediated elasticity of stress fibres. Nat. Commun. **8** , 15817. (10.1038/ncomms15817) 28604737 PMC5477492

[88] McMahon TA . 1984 Muscles, reflexes, and locomotion. Princeton University Press. (10.1515/9780691221540)

[89] Arslan YZ , Karabulut D , Ortes F , Popovic MB . 2019 Exoskeletons, exomusculatures, exosuits: dynamic modeling and simulation. In Biomechatronics, (ed. MB Popovic ), pp. 305–331. London, UK/San Diego, CA: Elsevier.

[90] Regazzoni F , Dedè L , Quarteroni A . 2021 Active force generation in cardiac muscle cells: mathematical modeling and numerical simulation of the actin-myosin interaction. Vietnam J. Math. **49** , 87–118. (10.1007/s10013-020-00433-z) 34722731 PMC8549950

[91] Wakeling JM , Febrer-Nafría M , De Groote F . 2023 A review of the efforts to develop muscle and musculoskeletal models for biomechanics in the last 50 years. J. Biomech. **155** , 111657. (10.1016/j.jbiomech.2023.111657) 37285780

[92] Discher DE , Janmey P , Wang YL . 2005 Tissue cells feel and respond to the stiffness of their substrate. Science **310** , 1139–1143. (10.1126/science.1116995) 16293750

[93] Janmey PA , Fletcher DA , Reinhart-King CA . 2020 Stiffness sensing by cells. Physiol. Rev. **100** , 695–724. (10.1152/physrev.00013.2019) 31751165 PMC7276923

[94] Chakraborty M *et al* . 2021 Mechanical stiffness controls dendritic cell metabolism and function. Cell Rep. **34** , 108609. (10.1016/j.celrep.2020.108609) 33440149

[95] Huxley AF . 1957 Muscle structure and theories of contraction. Prog. Biophys. Biophys. Chem **7** , 255–318. (10.1016/s0096-4174(18)30128-8) 13485191

[96] Huxley AF , Simmons RM . 1971 Proposed mechanism of force generation in striated muscle. Nature **233** , 533–538. (10.1038/233533a0) 4939977

[97] Powers JD , Malingen SA , Regnier M , Daniel TL . 2021 The sliding filament theory since Andrew Huxley: multiscale and multidisciplinary muscle research. Annu. Rev. Biophys. **50** , 373–400. (10.1146/annurev-biophys-110320-062613) 33637009

[98] Moreo P , García-Aznar JM , Doblaré M . 2008 Modeling mechanosensing and its effect on the migration and proliferation of adherent cells. Acta Biomater. **4** , 613–621. (10.1016/j.actbio.2007.10.014) 18180207

[99] Borau C , Kamm RD , García-Aznar JM . 2014 A time-dependent phenomenological model for cell mechano-sensing. Biomech. Model. Mechanobiol. **13** , 451–462. (10.1007/s10237-013-0508-x) 23783520 PMC4086324

[100] Cao X , Lin Y , Driscoll TP , Franco-Barraza J , Cukierman E , Mauck RL , Shenoy VB . 2015 A chemomechanical model of matrix and nuclear rigidity regulation of focal adhesion size. Biophys. J. **109** , 1807–1817. (10.1016/j.bpj.2015.08.048) 26536258 PMC4643201

[101] Alisafaei F , Jokhun DS , Shivashankar GV , Shenoy VB . 2019 Regulation of nuclear architecture, mechanics, and nucleocytoplasmic shuttling of epigenetic factors by cell geometric constraints. Proc. Natl Acad. Sci. USA **116** , 13 200–13 209. (10.1073/pnas.1902035116) PMC6613080 31209017

[102] Sun SY Zhang H Fang W Chen X Li B Feng XQ . 2022 Bio-chemo-mechanical coupling models of soft biological materials: a review. Adv. Appl. Mech **55** , 309–392. (10.1016/bs.aams.2022.05.004)

[103] Senthilkumar I , Howley E , McEvoy E . 2022 Thermodynamically-motivated chemo-mechanical models and multicellular simulation to provide new insight into active cell and tumour remodelling. Exp. Cell Res. **419** , 113317. (10.1016/j.yexcr.2022.113317) 36028058

[104] Sidhaye J , Norden C . 2017 Concerted action of neuroepithelial basal shrinkage and active epithelial migration ensures efficient optic cup morphogenesis. Elife **6** , e22689. (10.7554/eLife.22689) 28372636 PMC5380436

[105] Tallinen T , Chung JY , Rousseau F , Girard N , Lefèvre J , Mahadevan L . 2016 On the growth and form of cortical convolutions. Nat. Phys. **12** , 588–593. (10.1038/nphys3632)

[106] Grainge CL , Lau LCK , Ward JA , Dulay V , Lahiff G , Wilson S , Holgate S , Davies DE , Howarth PH . 2011 Effect of bronchoconstriction on airway remodeling in asthma. N. Engl. J. Med. **364** , 2006–2015. (10.1056/NEJMoa1014350) 21612469

[107] Park JA *et al* . 2015 Unjamming and cell shape in the asthmatic airway epithelium. Nat. Mater. **14** , 1040–1048. (10.1038/nmat4357) 26237129 PMC4666305

[108] Muñoz JJ , Albo S . 2013 Physiology-based model of cell viscoelasticity. Phys. Rev. E. Stat. Nonlin. Soft Matter Phys. **88** , 012708. (10.1103/PhysRevE.88.012708) 23944493

[109] Mosaffa P , Rodríguez-Ferran A , Muñoz JJ . 2018 Hybrid cell-centred/vertex model for multicellular systems with equilibrium-preserving remodelling. Int. J. Numer. Method. Biomed. Eng. **34** , e2928. (10.1002/cnm.2928) 28898926

[110] Ioannou F , Dawi MA , Tetley RJ , Mao Y , Muñoz JJ . 2020 Development of a new 3D hybrid model for epithelia morphogenesis. Front. Bioeng. Biotechnol. **8** , 405. (10.3389/fbioe.2020.00405) 32432102 PMC7214536

[111] Asadipour N , Trepat X , Muñoz JJ . 2016 Porous-based rheological model for tissue fluidisation. J. Mech. Phys. Solids **96** , 535–549. (10.1016/j.jmps.2016.07.002)

[112] Mosaffa P , Asadipour N , Millán D , Rodríguez-Ferran A , J Muñoz J . 2015 Cell-centred model for the simulation of curved cellular monolayers. Comp. Part. Mech. **2** , 359–370. (10.1007/s40571-015-0043-x)

[113] Muñoz JJ , Dingle M , Wenzel M . 2018 Mechanical oscillations in biological tissues as a result of delayed rest-length changes. Phys. Rev. E **98** , 052409. (10.1103/PhysRevE.98.052409)

[114] Solon J , Kaya-Copur A , Colombelli J , Brunner D . 2009 Pulsed forces timed by a ratchet-like mechanism drive directed tissue movement during dorsal closure. Cell **137** , 1331–1342. (10.1016/j.cell.2009.03.050) 19563762

[115] Heydari T , Heidari M , Mashinchian O , Wojcik M , Xu K , Dalby MJ , Mahmoudi M , Ejtehadi MR . 2017 Development of a virtual cell model to predict cell response to substrate topography. ACS Nano **11** , 9084–9092. (10.1021/acsnano.7b03732) 28742318

[116] McEvoy E et al . 2022 Feedback between mechanosensitive signaling and active forces governs endothelial junction integrity. Nat. Commun. **13** , 7089. (10.1038/s41467-022-34701-y) 36402771 PMC9675837

[117] Elosegui-Artola A , Oria R , Chen Y , Kosmalska A , Pérez-González C , Castro N , Zhu C , Trepat X , Roca-Cusachs P . 2016 Mechanical regulation of a molecular clutch defines force transmission and transduction in response to matrix rigidity. Nat. Cell Biol. **18** , 540–548. (10.1038/ncb3336) 27065098

[118] Bennett M , Cantini M , Reboud J , Cooper JM , Roca-Cusachs P , Salmeron-Sanchez M . 2018 Molecular clutch drives cell response to surface viscosity. Proc. Natl Acad. Sci. USA **115** , 1192–1197. (10.1073/pnas.1710653115) 29358406 PMC5819391

[119] Andreu I *et al* . 2021 The force loading rate drives cell mechanosensing through both reinforcement and cytoskeletal softening. Nat. Commun. **12** , 4229. (10.1038/s41467-021-24383-3) 34244477 PMC8270983

[120] McEvoy E , Shishvan SS , Deshpande VS , McGarry JP . 2018 Thermodynamic modeling of the statistics of cell spreading on ligand-coated elastic substrates. Biophys. J. **115** , 2451–2460. (10.1016/j.bpj.2018.11.007) 30527450 PMC6301989

[121] Shishvan SS , Vigliotti A , Deshpande VS . 2018 The homeostatic ensemble for cells. Biomech. Model. Mechanobiol. **17** , 1631–1662. (10.1007/s10237-018-1048-1) 29987699

[122] McEvoy E , Deshpande VS , McGarry P . 2019 Transient active force generation and stress fibre remodelling in cells under cyclic loading. Biomech. Model. Mechanobiol. **18** , 921–937. (10.1007/s10237-019-01121-9) 30783833

[123] Wortel IMN , Niculescu I , Kolijn PM , Gov NS , de Boer RJ , Textor J . 2021 Local actin dynamics couple speed and persistence in a cellular Potts model of cell migration. Biophys. J. **120** , 2609–2622. (10.1016/j.bpj.2021.04.036) 34022237 PMC8390880

[124] Hirway SU , Lemmon CA , Weinberg SH . 2021 Multicellular mechanochemical hybrid cellular Potts model of tissue formation during epithelial‐mesenchymal transition. Comp. Sys. Onco. **1** , e1031. (10.1002/cso2.1031)

[125] Odagiri K , Fujisaki H , Takada H , Ogawa R . 2022 Numerical simulation using cellular Potts model for wound closure with ATP release and the Mechanobioligical effects. arXiv See https://arxiv.org/pdf/2209.01354.pdf.

[126] Fletcher AG , Osterfield M , Baker RE , Shvartsman SY . 2014 Vertex models of epithelial morphogenesis. Biophys. J. **106** , 2291–2304. (10.1016/j.bpj.2013.11.4498) 24896108 PMC4052277

[127] Alt S , Ganguly P , Salbreux G . 2017 Vertex models: from cell mechanics to tissue morphogenesis. Phil. Trans. R. Soc. Lond. B. **372** , 20150520. (10.1098/rstb.2015.0520) 28348254 PMC5379026

[128] Li B , Sun SX . 2014 Coherent motions in confluent cell monolayer sheets. Biophys. J. **107** , 1532–1541. (10.1016/j.bpj.2014.08.006) 25296305 PMC4190608

[129] Bi D , Yang X , Marchetti MC , Manning ML . 2016 Motility-driven glass and jamming transitions in biological tissues. Phys. Rev. X **6** , 021011. (10.1103/PhysRevX.6.021011) 28966874 PMC5619672

[130] Giavazzi F , Paoluzzi M , Macchi M , Bi D , Scita G , Manning ML , Cerbino R , Marchetti MC . 2018 Flocking transitions in confluent tissues. Soft Matter **14** , 3471–3477. (10.1039/c8sm00126j) 29693694 PMC5995478

